# Logic Analysis of How the Emergency Management Legal System Used to Deal with Public Emerging Infectious Diseases under Balancing of Competing Interests—The Case of COVID-19

**DOI:** 10.3390/healthcare9070857

**Published:** 2021-07-06

**Authors:** Chao Zhang, Lei Hong, Ning Ma, Guohui Sun

**Affiliations:** 1 School of Law, Beijing Institute of Technology, Beijing 100081, China; zhangchao01@bjut.edu.cn; 2 Publicity Department of Party Committee, Beijing University of Technology, Beijing 100124, China; 3 BlueFocus Intelligent Communications Group Co., Ltd., Beijing 100015, China; lei.hong@Bluefocus.com; 4 Graduate School, Communication University of China, Beijing 100024, China; 5 Beijing Key Laboratory of Environment and Viral Oncology, College of Life Science and Chemistry, Faculty of Environment and Life, Beijing University of Technology, Beijing 100124, China

**Keywords:** emergency management legal system, public emerging infectious diseases, COVID-19, administrative coercive measures, balancing of competing interests

## Abstract

Development of measures for mitigating public emerging infectious diseases is now a focal point for emergency management legal systems. COVID-19 prevention and containment policies can be considered under the core goal of social and individual interests. In this study we analyzed the complexity between individual and public interests as they conflict when implementing disease preventative measures on an epidemic scale. The analysis was used to explore this complex landscape of conflicting social, public, and legal interests to quantify the potential benefits of public acceptance. Here we use the large-scale COVID-19 epidemic backdrop to examine legal norms of the emergency management legal framework. We find that the implementation of emergency management legal system measures involves the resolution of both direct and indirect conflicts of interest among public groups, individual groups, and various subsets of each. When competing interests are not balanced, optimal policies cannot be achieved to serve and safeguard shared social and community stability, whereas effective social outcomes are obtainable through the development of targeted policies as defined within the emergency management legal system. A balanced legal framework in regards to emergency management legal norms can more effectively serve to mitigate and prevent the continued spread of emerging infectious diseases. Further developing innovative procedural mechanisms as a means to ensure emergency response intervention should take into account the weighted interest of the different social parties to determine priorities and aims to protect legitimate public interests.

## 1. The Value of Balancing Competing Interests in the Emergency Management Legal System

The emergency management legal system for public emerging infectious disease prevention plays an important role, particularly in the fight against the coronavirus disease 2019 (COVID-19) pandemic, as it relates to legal issues directed at public emerging infectious disease prevention. Greater concern and discussion has been a societal and academic focus since the beginning of the pandemic. Several countries and regions have activated the emergency management of emerging public health events to combat the COVID-19 pandemic. Experts and scholars in health law and public health management research have provided opinions on the legal issues during the emergency management period. From current research to legal regulations to policies regarding emerging public health emergency events management measures, individuals are fundamentally focused on laws associated with emergency management and their application, and less so on the related associations involved within the basis of the balancing of emergency management interests of emerging public health events. 

### 1.1. The Paramount Goals of Emergency Management Legal Systems in Safeguarding Shared Social Community Interests

Scholars such as Fu et al. assume that in a modern society functioning under the rule of law, to prevent major emergencies from causing enormous impacts on people’s lives and resulting in complete civil disorder, administrative emergency powers and formal emergency laws must be used to change all forms of social relations when dealing with such emergencies, thereby ensuring that the harm caused by them is efficiently regulated or removed; that normal development, daily life, and legal order are restored; and that the legitimate rights of the public are protected [1]. Scholars such as Zeng and Dong have pointed out that the emergency management legal system is unique, as officials under this system are not required to obey common standards in the exercise of administrative powers and can instead execute their tasks in compliance with special legal procedures. In addition, such systems typically pay greater attention to the protection of public interests and stress the preservation of civil rights [2]. The above-mentioned scholars have further pointed out that “social order”, “social relations”, “public interests”, and “citizens’ legitimate rights and interests” by definition fall within the scope of interests of the emergency management legal system [1,2,3].

### 1.2. Function of the Balancing of Competing Interests of the Emergency Management Legal System

An emergency management legal system needs to coordinate and weigh both public interests and the interests of other subjects at a particular time and in a particular environment. Public interests are not abstract and out of reach, but occur in various forms or states on different occasions [4]. For example, in terms of labor law and consumer protection law, public interests refer specifically to the interests of the socially weak; in environmental laws and natural resources laws, public interests refer mostly to the rational conservation and use of community-owned resources; concerning criminal laws and laws regarding public security, the general public interest centers on the peace and protection of the social order; and in terms of laws regarding health and the prevention and control of public emerging infectious diseases, public interests refer to public health [5,6,7]. Public interests thus have the characteristics of integrity and universality in scope and content, while individual interest is partial and special [8]. For example, laws regarding the prevention and control of public emerging infectious diseases strictly regulate the treatment of patients and carriers of public emerging infectious diseases not only because the diseases can cause grave harm to patients and the carriers of pathogens and the treatment of infectious patients is an interest of considerable importance, but also because infectious carriers have the potential to cause harm to non-specific groups. The personal interests of such threatened unspecified people are socially common, and thus no longer constitute individual interest but, rather, are a matter of public interests [9,10]. Since the advent of modern times, Western scholars have discussed the application of interests balancing in the field of law. The German scholar Heck regarded the balancing of competing interests as an important aspect of his scholarship. Furthermore, Bentham from the UK, Jhering from Germany, Pound from the United States, and various other Western scholars made inspiring contributions in discussing the goal of balancing legal interests [11]. These contributions have provided theoretical and methodological guidelines regarding the balancing of competing interests in the emergency management legal system, with the overarching view being that the law should serve those interests that are most important and of the greatest priority. Furthermore, achieving reasonably fair objectives will only require certain groups to pay minimal costs [12]. The American jurist Bodenheimer proposed that one of the main functions of the law is to adjust and reconcile various conflicting interests, whether they are personal interest or social interests, and that such adjustments must be promulgated to a certain extent only by evaluating the importance of various interests and providing general rules for said adjustments to resolve conflicts of interest in a standardized manner [13]. In the process of addressing the various conflicts of interest, the important objective of the law is to give priority to safeguarding public interests. To paraphrase Professor Zhang, the formulation of the law should properly weigh the interests of the various parties involved and ensure the realization of the collective rationale, even while bearing in mind that improper restraints on individual rationality could result in collective irrationality [14].

**Figure 1:**
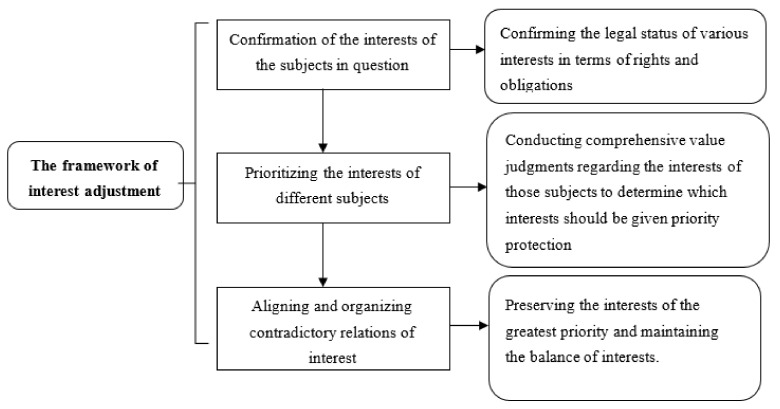
Framework of interest adjustment of the emergency management legal system.

Based on this idea, we can generally separate the benefits of the emergency management legal systems, the contents of which include both the emergency management legal system rights and responsibilities. An emergency management legal system should also give priority to public interests via the means through which it coordinates conflicting interests between different subjects, while also reducing the risks of sacrificing other possible interests and reduce resistance to a minimum. Specifically, such an interest adjustment is mainly composed of three parts, as shown in Figure 1. First, it entails the confirmation of the interests of the subjects in question—that is, confirming the legal status of various interests in terms of rights and obligations. Second, it requires prioritizing the interests of different subjects by conducting comprehensive value judgments regarding the interests of those subjects to determine which interests should be given priority protection and/or which vulnerable groups should be given preferential protection. Third, it entails aligning and organizing contradictory relations of interests and preserving the interests of the greatest priority, such as public interests, protecting the weak, reducing the risk of damage, and maintaining the balance of competing interests. When discrepancies between different values occur, a comprehensive consideration must be applied based on the actual conditions to determine which interests must be protected, instead of applying a finite and absolute guideline for evaluation.

## 2. Conflicts of Interest in Addressing Public Emerging Infectious Diseases

Based on the alignment of interests within an emergency management legal system, in taking action to prevent and control public emerging infectious diseases, it is important to discuss the causes for the relations of interests that might be involved in the execution of the emergency management legal system measures, in particular possible conflicts of interest.

### 2.1. Explicit Conflicts of Interest

One explicit conflict of interest involved in the prevention and treatment of public emerging infectious diseases is the conflict between infected patients, including asymptomatic carriers and suspected patients (Taking COVID-19 as an example, on 5 February 2020, the National Health Commission issued the “New Coronavirus Diagnosis and Treatment Program (Trial Version 5)”, making it clear that the current source of infection primarily consists of patients with coronavirus infections, and that asymptomatic infections may also be a source of infection.), and healthy citizens and individuals. The interests of these groups conflict because of the direct potential for infection. The most important method for the prevention, control, elimination of public emerging infectious disease occurrences, and protection of public health, is to control the sources of infection and the compulsory isolation and treatment of infectious patients. Infected patients always hope that the compulsory management measures imposed on them can be reduced to a minimum to lessen the loss of personal freedom. Since a public emerging infectious disease is particularly liable to cause infections, people can experience deep anxiety when confronted with unknown public emerging infectious diseases and may want to make a clean break from the real world, which can, in turn, lead to a social crisis. (People are not always rational and inclusive when faced with public emerging infectious diseases. For example, people infected with leprosy were historically dealt with using non-rational measures, including very cruel persecution, with some being burned to death or even buried alive or drowned. Leprosy-infected patients were viewed as already being dead. It was said that there would thus be a funeral ceremony before such a patient before the patient entered quarantine. Then, after their release from quarantine, the outdoor activities of such patients were limited, and they had to ring bells if they went out, so that others could avoid them. In medieval Europe, there were often rumors that people transported leprosy patients in large numbers to the sea and then drowned them there. The most humane way to deal with leprosy patients, meanwhile, was generally to expel them from the community and let them die (Sun, 2010)).

### 2.2. Implicit Conflicts of Interest

Many hidden conflicts of interest may be involved in the prevention and treatment of public emerging infectious diseases, with such conflicts of interest themselves mainly consisting of two types: conflicts between individual interest and public interests and conflicts between the interests of various other parties. Since the main task of an emergency management legal system is to safeguard public interests, these two types of implicit conflicts of interest have become important issues that require coordination and resolution under the law.

#### 2.2.1. Conflicts between Individual Interest and Public Interests

Many hidden conflicts of interest may be involved in the prevention and treatment of public emerging infectious diseases, with such conflicts of interest themselves mainly consisting of two types: conflicts between individual interest and public interests and conflicts with various third parties. Since the main task of an emergency management legal system is to safeguard public interests, these two types of implicit conflicts of interest have become important issues that require coordination and resolution under the law.

There are two major types of conflicts between individual interest and public interests: conflicts between infectious patients and public interests and conflicts between uninfected patients and public interests. In the first type of conflict of interests, the interests of infectious patients and public interests will form a contradictory relationship. Taking the right of privacy of infectious patients as an example, in the absence of a public emerging infectious disease epidemic, citizens’ right to privacy is of course protected according to law. Nevertheless, when an epidemic occurs, the right to privacy of infected patients can become an obstacle to public health and safety, and, to the extent that we still need to serve the greatest number of individual interests possible, inevitably threaten public safety. Therefore, the individual interest of infectious patient needs to make concessions to public interests, and certain waivers of rights may even be required. Information on the causes of infection, physical status, living habits, criminal behaviors, past medical histories, family histories, and genetic histories of infectious patients should, relatedly, be disclosed to medical personnel in their undertaking of medical activities [15], and even the trajectory of confirmed cases can be disclosed to the public according to the needs of epidemic prevention and control. In the second type of conflicts of interest, the interests of individuals, such as those of individual people or enterprises, and public interests will also have a contradictory relationship. When an epidemic occurs, governmental authorities will take corresponding compulsory measures in compliance with the applicable emergency management laws and regulations. In addition to the anti-epidemic compulsory measures directly affecting infectious patients, such as the mandatory quarantine and treatment of patients, there will also be mandatory measures for ordinary citizens. For instance, the Wuhan COVID-19 Prevention and Control Headquarters issued a notice on 23 January 2020, that, from that day at 10:23 onwards, Wuhan city buses, subways, ferries, and long-distance passenger trains were required to suspend operations, with airports and train stations offering transportation out of Wuhan being temporarily closed. Citizens were not allowed to leave Wuhan without extenuating circumstances [16]. As a result, many travel arrangements had to be canceled. In addition, to prevent the spread of the epidemic, the State Council extended the Spring Festival holiday, impacting production for many enterprises. (The Notice of the General Office of the State Council on Extending the 2020 Spring Festival Holiday stipulated that the 2020 Spring Festival holiday would be extended to 2 February and normal work would begin on 3 February. When the Spring Festival holiday ended, the epidemic situation in some places had not been controlled. The relevant government departments issued a notice recommending that employees work from home, and that enterprises whose employees could not work from home should encourage flexible working hours or flexible working hour systems. Under the influence of various restrictive measures, the production and operation activities of enterprises were restricted and their economic interests were affected). The rights of individual entities, including residents and corporations, were impacted in this way by the introduction of emergency response measures. In summary, during the period of prevention and control of emerging infections to the public, individual interests of members of a society and public interests will overlap in some ways; however, contradictions and conflicts will inevitably result.

#### 2.2.2. Complex Conflicts between the Interests of Different Types of Subjects

An emergency management legal system needs to face conflicts of interest among different types of subjects. These complicated contradictions between the interests of various parties are inseparable from the complexities of common interests and profound and far-reaching effects of public emerging infectious diseases. Since there are many conflicts of interest between different types of subjects, only a few typical ones are listed here. One type consists of conflicts between individual interests and collective interests (partial public interests). Throughout the public emerging infectious disease prevention and control phase of an epidemic, the autonomy of villages and villagers plays an important role in the mobilization of efforts to prevent public emerging infectious disease outbreaks, ensure mass control of communities, and prevent further infections. Relatedly, during the implementation of “internal anti-proliferation and external anti-export” quarantine measures, conflicts between individual interests and collective interests will be triggered. For example, media reports revealed that after the Spring Festival, many communities in Beijing prohibited people from entering the city. Only after the Civil Affairs Department intervened was the problem resolved [17]. The second type of conflict consists of conflicts of public interest among varying regions. For example, during the period of prevention and control of COVID-19, there was a shortage of personal protective equipment such as medical protective clothing and masks. Although the shortage was ultimately alleviated through various efforts, the problem remained prominent in most parts of the country. During this period, the Dali City Government of Yunnan Province intercepted medical materials sent to Chengdu, Sichuan, Chongqing, Huangshi City, Hubei Province (epidemic-stricken area), and similar places, causing widespread concern in society. The person responsible was later punished accordingly [18], but this incident exposed the conflicts of public interest among the various regions. The third type of conflict consists of conflicts between group interests and public interests. For example, in the “face mask price spike” incident, due to the expansion and severity of the COVID-19 epidemic exceeding expectations, mask-wearing behavior changed from an advocated practice to a mandatory one, which resulted in an increase in mask prices in some pharmacies. The interests of pharmaceutical retailers and the public were thus in conflict. The public wanted to be able to buy protective equipment at a low price during the outbreak, but pharmaceutical retailers were reluctant to sacrifice their economic interests by selling at losses. Nonetheless, pharmaceutical companies soon found it difficult to regularly purchase masks due to the high costs. Relatedly, if they then sold masks at a lower price, they would incur losses. Conversely, if they increased the prices, they could easily be punished by customer complaints or the State Administration for Industry and Commerce (SAIC). Retailers thus had to choose whether or not to purchase and sell the goods [19].

**Figure 2:**
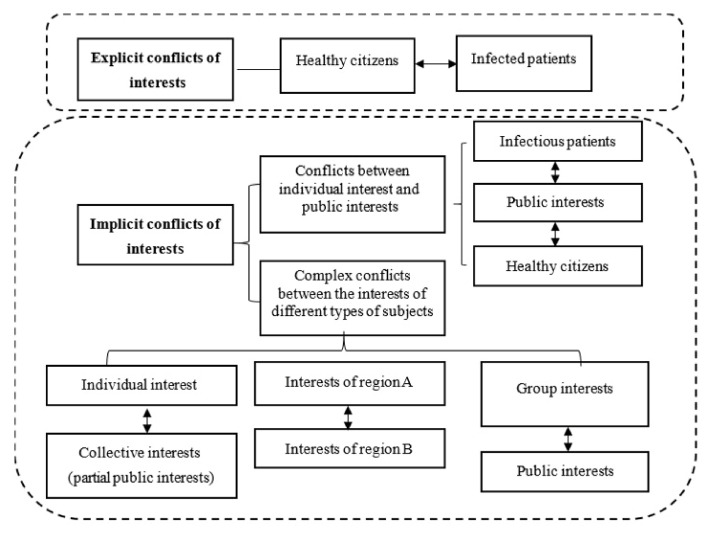
Conflicts of interest addressing public emerging infectious diseases.

In addition, during prevention and control efforts for outbreaks of public emerging infectious diseases, there may be contradictions and conflicts inside some particular public interest groups. It is not difficult to see from the preceding analysis that the interests of society are rooted to a certain degree in production relations; that the realization of interests is therefore done under certain conditions with limited resources, presenting issues of finiteness and conditionality when confronted by the needs of different interest groups, giving rise to conflicts of interest as a result of the differing goals as pursued by the related parties [20], as shown in Figure 2.

## 3. Emergency Management Legal System for Epidemic Prevention and Control Regulations for the Balance of Competing Interests

**Figure 3:**
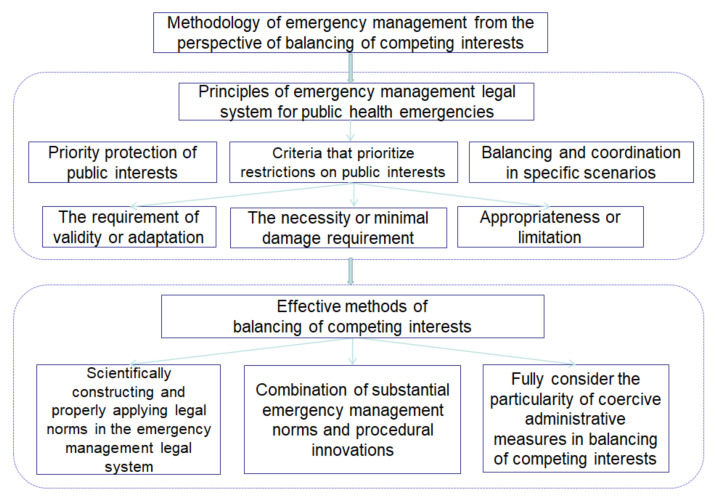
Methodology of emergency management from the perspective of balancing competing interests.

To sum up, there are two primary types of conflicts of interest: overt conflicts of interest and implicit conflicts of interest in the prevention and control of public infection. We need to use a complete methodology system to strengthen the construction of the emergency management legal system to balance competing interests, as shown in Figure 3.

### 3.1. The Rationality of Balancing Interests

#### 3.1.1. Prioritizing the Protection of Public Interests

The emergency management legal system laws provide a normative system formed to regulate social relations under extenuating circumstances to pursue the public’s best interests. Civil laws and other areas of private law focus on the protection of individual interests. In a case that involves avoiding damage to individual interest or minimizing damage, priority should be given to protecting public interests.

The need to preserve social stability and growth requires safeguarding public interests. The sustainability and growth of society depend on the security of public interests. To paraphrase Machiavelli, it is not the pursuit of personal interest but common interests that make city-states powerful [21]. If public interests are not given priority in terms of protection, they will be at a disadvantage when conflicting with individual interests, leading to further social threats.

#### 3.1.2. Analyzing the Essence of Relationship Interests

Public interests and personal interests are essentially the same. The essential attributes of an individual and the relationship between community members and the community show that public interests and private interests are inherently consistent. When an individual believes that he is pursuing his interest, he is also pursuing public interests [22,23]. Some scholars have also pointed out that, in addition to individual interests, public interests and social group interests also have internal consistency. In fact, in regards to the cause of public interests, the compositions of groups of people in various forms effectively generate public interest, the fundamental purpose of which is to serve individuals as members of the community [24,25]. Public interests are not a simple summary of individual interests. Rather, the two types of interests are both opposing and unifying and generally show a mutually symbiotic relationship.

#### 3.1.3. Analysis of the Nature of an Emergency Management Legal System

The nature of an emergency management legal system determines the priority position of the protection of public interests. Government leadership is a basic means of providing crisis management [26]. The main task of an emergency management legal system is to explain the standardization of special administrative procedures in an emergency, so that the emergency management legal system can gradually progress towards a streamlined, institutionalized, and legalized track, which is an important basis for the emergency management legal system. In a modern country ruled by law, to prevent the entire country from falling into complete civil disorder as a result of a major emergency, it is necessary to use administrative emergency powers and implement emergency legal regulations supporting the system to adjust various social relations during public emergencies, thereby effectively controlling and eliminating the harm caused by emergencies and safeguarding public interests [27].

#### 3.1.4. Prioritizing Social Public Interests and the Limitations

The mutual relationship between public interests and private interests dictates that the priority of neither type of interest is absolute and that public interests must therefore be fairly limited. The following criteria must be followed when affirming the importance of public interests.

Efforts aimed at the protection of public interests need to follow the principle of law reservation. As far as the law reservation requirements concerning the priority of public interests are concerned, it specifically means that any norms other than the law shall not stipulate the priority norms of public interests [7]. For example, Article 8 of China’s current Legislation Law stipulates that compulsory measures and fines for people that limit their liberty can only be governed by statute. Therefore, to safeguard public health and safety needs by quarantining patients with COVID-19, such quarantining must be allowed by the Infectious Disease Control Law rather than by other legal regulations. Unless other legal authorizations exist, the relevant provisions of the Emergency Regulations for Public Health Emergencies are also legally authorized. This is necessary to reduce defects such as abstract vagueness or loopholes in the regulations themselves that could result in the infringement of the rights of other subjects in practice.

Actions aimed at the protection of public interests need to abide by the principle of proportionality. During the prevention and treatment of public emerging infectious diseases, the emergency management measures taken by administrative organs will inevitably influence public interests and the interests of other parties, while the principle of proportionality provides basic guidelines for balancing these two types of interests. Three important factors are informing the use of the principle of proportionality [28]: (The essence of the principle of proportionality is determining the balancing between administrative priorities and administrative means in the event of a clash between administrative powers and civil rights. In such cases, the administrative power may cause certain damages to the rights of administrative counterparts when it pursues the administrative goals. In order to avoid constitutional rights from being unfairly violated by governmental authorities, it is important to handle citizens’ rights in a fair manner. The core of the principle of proportionality is preventing administrative powers from excessively infringing upon citizens’ rights, stressing that they “do not shoot birds with cannons.” (Ma, 2000)).

- The requirement of validity or adaptation;
- The necessity or minimal damage requirement;
- Appropriateness or limitation.

Protecting public interests also requires following the principle of reasonable compensation. That is, that the loss caused by the maintenance of public interests to the interests of other subjects is minimized through administrative compensation, or even that all losses be covered through such compensation, but it emphasizes reasonable compensation [29]. For example, during the prevention and control of public emerging infectious diseases, the state should provide compensation for medical protective equipment, such as masks and protective clothing, to the various organizations or individuals from which such equipment is requisitioned.

#### 3.1.5. Balancing and Coordination of Conflicts of Interest in Specific Instances

The emergency management legal system measures should follow the goal of safeguarding public interests, while also reducing any infringement on the interests of other parties. When there are conflicts among the interests of various parties, the reasons for those conflicts of interest should be fully considered. The administrative agencies should resolve outstanding problems and contradictions, basing their actions on the principles of lawfulness, rationality, and due process, to prevent the sacrifice of local or group interests for partial interests, specifically the sacrifice of the legitimate rights and interests of individual citizens. During the prevention and control period of a public emerging infectious disease, there are always delicate and complex conflicts of interest. For example, during the outbreak of COVID-19 in Wuhan, there was a severe shortage of ambulances. At the same time, both COVID-19 and non-COVID-19 patients, such as those suffering from strokes, myocardial infarctions, etc., needed ambulances. Determining the value of different patients’ lives is difficult [30], and in the face of complex conflicts of interest and difficult choices, we may not be able to rely on a simple pre-determined authoritative ranking of different interests. In such circumstances, strict legal processes aimed at protecting legitimate interests, regardless of whether they are public interests or private interests, have an especially important role to play and have become the primary method for determining the priority of public interests [31].

### 3.2. Effective Methods of Balancing Competing Interests

While safeguarding a country’s overall economic and social development interests and fully safeguarding public interests in compliance with the law, the adoption of the principles of the rule of law and the scientific methods is also required for an emergency management legal system to fully play its role in addressing various conflicts of interest.

#### 3.2.1. Scientific Construction and the Proper Application of Legal Norms in the Emergency Management Legal System

An emergency management legal system should unify active prevention efforts with scientific emergency response efforts. Through the guidance of laws and regulations, such systems are encouraged to shift the focus of the emergency management legal system from emergency response to crisis prevention and emergency support. For example, in 2003 after years of SARS outbreaks, there was still no scientific system of preventive measures against such outbreaks, and the relevant departments did not place effective regulations on eating and trading wildlife, a failure which in turn may have contributed to the recent COVID-19 outbreak (the exact origin of the public emerging infectious disease remains contested) and resulted in a “human-instigated disaster”. Once an outbreak of a public emerging infectious disease occurs, scientific and reasonable emergency management legal system measures must be taken to minimize the risks from the public emerging infectious disease, and to improve the system contents of emergency alertness, information disclosure, and emergency recovery and reconstruction.

The prevention and control of outbreaks of public emerging infectious diseases is a complex project involving a wide range of fields and multiple subjects. An emergency management legal system should seek to align the government’s leading role and public participation. Government has the responsibility to deal with epidemic outbreaks of public emerging infectious diseases and other environmental and social emergencies. Given that public health and safety is a typical public good, it is difficult to regulate through market behavior; therefore, the government is the leader in handling public health emergencies. While the government plays a leading role, however, it cannot do so without the support of public participation. On the one hand, then, an emergency management legal system needs to establish sufficient emergency management legal system powers for administrative agencies. On the other hand, it should also provide institutional conditions for public involvement, encourage the public to engage in policy formulation and the activities of the system, and mobilize public forces to actively participate in the process of public emerging infectious disease prevention and control to reduce the resistance of the government in the process of emergency resource scheduling and to align the leading role of the government with the active participation of the public at the institutional level.

An emergency management legal system should seek to align the state leadership and the comprehensive responses of all parties. To prevent tension between the interests of the various parties during an emergency response phase, such as that for a public emerging infectious disease, it is necessary to plan the organizational system of emergency management at the level of the emergency management legal system to form a comprehensive system led by a fully unified country and the active cooperation of local authorities at all levels and relevant units, organizations, and individuals to resolve conflicts of interest between regions and groups in terms of organizational arrangements. In terms of the construction of emergency management organizations, developed Western countries generally set up a command center and a permanent comprehensive coordination department for crisis management at the central level that is responsible for coordinating, dispatching, directing, and mobilizing resources in various regions to respond to emergencies. These countries also collaborate with experts in various fields, develop long-term emergency strategies and plans from the height of national security, and set up corresponding departments at local levels [32].

#### 3.2.2. Combination of Substantial Emergency Management Legal System Norms and Procedural Advances

When applying significant norms, the relevant administrative sectors of the emergency management legal system should use innovative procedural mechanisms as the means of application to ensure emergency response interventions are acceptable and weigh the interests of the different parties to the greatest extent possible, specifically, the three following methods:

- Building an emergency management legal system launching mechanism with the participation of all the relevant interest groups. The government needs to fully exploit its position of authority to understand the needs of all the parties of interest to create a network that reflects diverse interests at the decision-making level, thereby allowing the actions of the emergency management legal system to extend to all parties in society and allowing for scientific judgments to be made; Exploring the mechanism of using industry experts to independently conduct benefit assessments. In the process of the prevention and treatment of public emerging infectious diseases, relevant departments within the emergency management legal system can organize medical experts in the prevention and treatment of public emerging infectious diseases who have no interests in common with those of all the parties involved into an expert group. Other public health or management experts in the field can also, if necessary, make professional assessments of upcoming emergency management legal system measures, render judgments on the possible impacts on various interests, and submit opinions and suggestions from objective positions; and
- Making use of the information disclosure system in the given emergency management legal system. In responding to outbreaks of public emerging infectious diseases, the timely and reliable release of information on outbreak prevention and control can not only eradicate rumors with credible information, thereby preserving social stability, but also completely protect the rights of the public, such as the right to be informed. Furthermore, providing information in this way also plays an important role in reducing the loss of life and property among all parties. An information disclosure system has a certain degree of flexibility and adaptability, which is useful for resolving various conflicts of interest. In the process of preventing and controlling outbreaks of public emerging infectious diseases, one major obstacle that can cause difficulties is information asymmetry, which can result in the emergency management legal system behaviors being increasingly involved in mediating intertwined interests, which can, in turn, result in conflicts. In previous emergencies, some related administrative departments did not disclose the information promptly and adopted a “one size fits all” approach to their efforts; as a result, the opposite outcome was achieved. The public release of information relevant to an outbreak of public emerging infectious diseases not only provides information to the public, but, most significantly, unifies all parties, incorporates social resources, and effectively assists the government by encouraging public engagement in a collective response to the epidemic [33]. Given the need to resolve various social crises promptly during an outbreak of emerging infectious diseases, it is necessary to use the information disclosure system flexibly to effectively resolve information asymmetry-related conflicts of interest, to ease social conflicts, and to ensure the smooth implementation of the emergency management legal system measures [34,35].
- In terms of minimizing peak logistical pressures, the optimum intervention may not be equivalent to one which minimizes the total epidemic size or, most certainly, minimizing direct social or economic impact from the intervention itself [36]. For this reason, the aims of a public health intervention policy must be clearly defined, with the intention that in the early phase of a pandemic sufficient resources can be allocated into characterizing the virus strain and measuring key epidemiological parameters as an essential foundation for decisions in developing the optimal mitigation strategy [37].

## 4. Conclusions

In this paper, we took a first step in introducing the value of balancing multiple competing interest groups into an otherwise standard law of conflict of interest model. This generalization is important in the context of the COVID-19 epidemic, since existing evidence demonstrates large differences in the considerations confronting disease prevention for the different groups. After providing a basic analysis of the dynamics of interests in the multiparty setting, we proceeded to an investigation of optimal policy, focusing in particular on the situational decisions facing policy makers between saving lives and maintaining social order and stability. Optimal uniform policy, which treats different group interests asymmetrically brought on by COVID-19, underscores the difficult choices confronting policy makers in the emergency management legal system to safeguard shared social and community interests. In our analysis of the functions for balancing competing interests, if the interests of one group are made the focus, then the impact on the remaining groups leads to greater instability.

The main result in fact is that more effective social outcomes are possible with targeted policies as defined by the management legal system. In dealing with the epidemic outbreak, coordinating differential policies with different interests can significantly improve policy compromises, enabling greater social and public acceptance. We also find that the majority of the achievable gains can be achieved by applying the law of conflict of interest with greater scientific application, targeting methods of interest and conflict measurements, and aggressively applying policies to manage the relation between public social and personal interests to the benefit of public health.

One issue we did not address is how policies can be implemented, considering a country’s population can be heterogeneous by group and involve various public and private elements. The first aspect of this issue is that voluntary behavioral changes may already achieve some of the objectives of the emergency management policies (use of face masks, voluntary social distancing, and remote work). The second aspect concerns implementation. While semi-targeted policies may have a higher rate of acceptance, stricter policies have to be identified as a form of protection for the whole of society and not to reduce the externalities they create on individual groups.

We view this paper as a first step towards the enrichment of the emergency management legal system norms and procedures intended as a tool for understanding and combating the COVID-19 epidemic, by introducing the conflicts and benefits of measuring accurately and balancing the weight of competing public and private interests. Our conclusion is that measured policies as constructed through the legal norms of emergency management can significantly improve social welfare, social stability, and social order. 

## Data Availability

Not applicable.
